# Inhibition of ferroptosis reverses heart failure with preserved ejection fraction in mice

**DOI:** 10.1186/s12967-023-04734-y

**Published:** 2024-02-24

**Authors:** Yixiao Xiong, Xin Liu, Ling Jiang, Tao Hao, Yanyan Wang, Tao Li

**Affiliations:** 1 Department of Anesthesiology, National-Local Joint Engineering Research Centre of Translational Medicine of Anesthesiology, West China Hospital, Sichuan University, No 37 Wainan Guoxue Road, Sichuan, 610041 China; 2 Laboratory of Mitochondria and Metabolism, West China Hospital, Sichuan University, Sichuan, 610041 China; 3 Department of Anesthesiology, West China Second Hospital of Sichuan University, Chengdu, 610041 Sichuan China; 4 Key Laboratory of Birth Defects and Related Diseases of Women and Children (Sichuan University), Ministry of Education, Sichuan University, Chengdu, 610041 Sichuan China; 5 Department of Gastroenterology, Chengdu Fifth People’s Hospital, No. 33 Mashi Street, Chengdu, 611130 Sichuan China; 6 Nursing Key Laboratory of Sichuan Province, West China Hospital, Sichuan University, No 37 Wainan Guoxue Road, Chengdu, 610041 Sichuan China

**Keywords:** Heart failure with preserved ejection fraction, Ferroptosis, Bioinformatics analysis, Ferrostatin-1, Deferiprone

## Abstract

**Background:**

Heart failure with preserved ejection fraction (HFpEF) accounts for approximately 50% of heart failure cases. The molecular mechanisms by which HFpEF leads to impaired diastolic function of the heart have not been clarified, nor have the drugs that target the clinical symptoms of HFpEF patients.

**Methods:**

HFpEF chip data (GSE180065) was downloaded from the National Center for Biotechnology Information (NCBI) database. Differentially expressed genes (DEGs) were filtered by the limma package in R and processed for GO and KEGG pathway analyses. Then, ferroptosis-related genes in HFpEF were identified by taking the intersection between DEGs and ferroptosis-related genes. CytoHubba and MCODE were used to screen ferroptosis-related hub DEGs in the protein–protein interaction (PPI) network. Establishment of a mouse HFpEF model to validate the transcript levels of ferroptosis-related hub DEGs and ferroptosis-related phenotypes. Transcript levels of ferroptosis-related hub DEGs and HFpEF phenotypic changes in the hearts of HFpEF mice were further examined after the use of ferroptosis inhibitors.

**Results:**

GO and KEGG enrichment analyses suggested that the DEGs in HFpEF were significantly enriched in ferroptosis-related pathways. A total of 24 ferroptosis-related DEGs were identified between the ferroptosis gene dataset and the DEGs. The established PPI network was further analyzed by CytoHubba and MCODE modules, and 11 ferroptosis-related hub DEGs in HFpEF were obtained. In animal experiments, HFpEF mice showed significant abnormal activation of ferroptosis. The expression trends of the 11 hub DEGs associated with ferroptosis, except for Cdh1, were consistent with the results of the bioinformatics analysis. Inhibition of ferroptosis alters the transcript levels of 11 ferroptosis-related hub DEGs and ameliorates HFpEF phenotypes.

**Conclusions:**

The present study contributes to a deeper understanding of the specific mechanisms by which ferroptosis is involved in the development of HFpEF and suggests that inhibition of ferroptosis may mitigate the progression of HFpEF. In addition, eleven hub genes were recognized as potential drug binding targets.

**Supplementary Information:**

The online version contains supplementary material available at 10.1186/s12967-023-04734-y.

## Introduction

Heart failure with preserved ejection fraction (HFpEF) is a chronic systemic syndrome characterized by an ejection fraction greater than 50% but with cardiac diastolic dysfunction [1]. Epidemiologic studies have shown that more than half of patients with heart failure have preserved ejection fraction, and this percentage increases over time [2]. Unlike heart failure with reduced ejection fraction (HFrEF), HFpEF is not a disease limited to the heart but involves multiple organs and complications. The prevalence of HFpEF is also increasing with increasing rates of obesity, hypertension and diabetes mellitus [2, 3]. Although the results of a phase III clinical trial showed that empagliflozin (SLGT2 inhibitor) reduced the risk of death due to cardiovascular disease exacerbation in HFpEF patients [4], there is still a lack of evidence-based evidence for the treatment of HFpEF [5]. Therefore, a better understanding of the underlying pathological mechanism is required for the development of new approaches for HFpEF treatment.

Ferroptosis, an oxidized, iron-dependent form of regulated cell death, was first described by Stockwell in 2012 and differs from apoptosis, necrosis and autophagy [6]. This unique programmed cell death is driven by iron-dependent phospholipid peroxidation, which leads to the accumulation of lipid reactive oxygen species (ROS), ultimately leading to cell membrane rupture [7]. Multiple cellular metabolic processes, including iron accumulation, mitochondrial activity, and lipid, amino acid, and carbohydrate metabolism, are involved in the regulation of ferroptosis [8]. Ferroptosis has been implicated in several types of pathological cell death associated with cardiovascular disease (i.e., heart failure, myocardial infarction, cardiomyopathy), cancer, acute renal failure, and other conditions [9–13]. Meanwhile, accumulated evidence has indicated that ferroptosis plays a vital role in the initiation and progression of HFrEF [14, 15], but little evidence shows how it affects HFpEF.

Currently, transcriptomic and microarray analyses have been widely used to identify new biomarkers in HFpEF [16]. In this study, we downloaded a microarray dataset of HFpEF to compare and analyze the original transcriptomic data. We also identified ferroptosis-associated hub genes through intersection with the ferroptosis dataset and protein‒protein interaction analysis. Then, we established a HFpEF mouse model and verified the expression of ferroptosis-related hub genes. More importantly, we found that inhibition of ferroptosis significantly alleviated HFpEF-associated clinical phenotypes, such as cardiac diastolic dysfunction and pulmonary congestion, and altered the expression of hub DEGs. These results provide new perspectives on potential therapeutic targets for HFpEF.

## Materials and methods

### Data information

The microarray profiles in GSE180065 were downloaded from the GEO database. The dataset involves 15 cardiac tissue samples, including 5 control samples, 5 samples with a high-fat diet (HFD)+ Nω-nitro-l-arginine methyl ester (L-NAME, 0.5 g/L dissolved in saline) modeling time of 5 weeks and 5 samples with HFD+L-NAME modeling time of 7 weeks. Five mice modeled with 5 weeks and five control group mice were selected for further bioinformatics analysis. In the original study, the HFpEF model was generated by a combination of a HFD and L-NAME for 5 weeks. The heart tissues were harvested for subsequent experiments.

### Differential expression analysis

Data processing was conducted using different packages of R (version: 4.3.0) under RStudio. Briefly, GSE180065_RAW.tar and its corresponding annotation platform GPL24247 were downloaded. The data in the control group and HL5W group were combined in the expression matrix. After normalization, differential expressed genes (DEGs) were identified using the “limma” package. Genes with a p value < 0.05 and |log2(fold-change)| ≥ 1 were considered DEGs. The obtained DEGs were then visualized using the R packages “ggplot” and “Heatmap”.

### Functional enrichment analysis

All DEGs were analyzed by Gene Ontology (GO) and Kyoto Encyclopedia of Genes and Genomes (KEGG) using the enrichGO and enrichKEGG functions in the R package “clusterProfiler”.

### Identification of ferroptosis‑related DEGs

The obtained ferroptosis-related DEGs were uploaded to the STRING online tool. The results were then visualized using Cytoscape to obtain protein–protein interaction (PPI) networks. To obtain the hub genes, the obtained PPI networks were further analyzed using MCODE and CytoHubba plugins in Cytoscape.

### Animals

C57BL/6J mice were purchased from Charles River. Mice were placed in an animal house on a 12 h light-dark cycle with unrestricted access to food (normal diet and high-fat diet) and water. L-NAME (0.5 g/l, Sigma-Aldrich) was adjusted to a pH of 7.4 and then added to the drinking water. Ferrostatin-1 (Med Chem Express) was dissolved in 0.1% DMSO and 99.9% corn oil at a concentration of 2 mg ml^−1^ and injected intraperitoneally at a dose of 2 mg kg^−1^ once daily for 2 weeks. Deferiprone (Med Chem Express) was dissolved in phosphate-buffered saline (PBS) and administered intraperitoneally at 100 mg kg^−1^ over 2 weeks.

### Exercise tolerance test

After allowing the mice to acclimatize on the small animal treadmill for 3 weeks, the experimental group of mice was tested for fatigue. The mice were acclimatized to the treadmill with a 20° incline for 4 min starting at a speed of 5 m min^−1^ and increasing by 2 m min^−1^ every two minutes thereafter until the mice were unable to resume running after 10 s of contact with the electrical stimulation grid. The running distance was recorded.

### Intraperitoneal glucose tolerance test

After 6 h of fasting, glucose (2 g kg^−1^) was administered intraperitoneally. Mouse tail blood glucose was measured before (0 min) and 15, 30, 45, 60 and 120 min after intraperitoneal injection.

### Conventional echocardiography and Doppler imaging

Echocardiographic measurements were performed under 1.5% isoflurane anesthesia, and the structure and function of the mouse heart were measured using a Vevo 3100 small animal high-resolution sonograph (Fujifilm Visual Sonics), which was swept with an MX550D probe (26–52 MHZ). The heart rate of the mice was maintained at 350–450 beats per minute throughout the ultrasound acquisition. The parameters collected included left ventricular ejection fraction, peak mitral Doppler flow velocity in early diastole, peak mitral Doppler flow velocity in late diastole, and peak tissue Doppler of mitral annular myocardial diastolic velocities in early diastole and early filling deceleration.

### Histology

The hearts were rapidly fixed overnight with 4% paraformaldehyde after isolation, embedded in paraffin and sectioned. Staining of heart sections using wheat germ agglutinin (WGA) and Masson’s trichrome (MT). Immunochemical staining of myocardial sections was performed using a CD31 primary antibody (1:2000, Cat. ab182981, Abcam). WGA results were observed using an Olympus IX83 microscope and quantitatively analyzed using ImageJ software 2.0. MT-stained and CD31 immunohistochemically stained sections were observed using a ZEISS Imager. A2 microscope. Tissue Prussian blue staining was performed using a Prussian blue staining kit (Cat. G1422, Solarbio) and observed using a ZEISS Imager. A2 microscope.

### IHC analysis

Heart myocardial tissues were isolated and fixed with 4% paraformaldehyde. After routine paraffin-embedded sections, the sections were deparaffinized and immersed in antigen repair solution and microwaved for 8 min, repeated three times. After cooling in a ventilated area, the slides were blocked by 5% bovine serum albumin (BSA) for 1 h and again washed 3 times with TBST. The slides were treated with 0.1% Triton X-100 for 10 min and then washed three times with TBST. Then the sections were incubated with F4/80 Polyclonal antibody (1:4000, Cat. 28463-1-AP, Proteintech) overnight. After incubation, slides were washed 3 times with TBST and incubated with goat anti-rabbit IgG (Alexa Fluor 594, 1:500; ab150080, Abcam) for 30 min at room temperature. The slides were washed 3 times with TBST and the slides were incubated with 4’,6-diamidino-2-phenylindole (DAPI). Images were visualized using a ZEISS Imager. A2 microscope microscope system and processed with ZEISS software.

### Measurement of ROS

To assess ROS production, the heart was carefully and quickly isolated, cut into 4.0 μm sections and placed on chilled microscope slides. The samples were incubated in physiological saline containing 10 µmol dihydroethidium (DHE; Cat. 104821-25-2, Sigma-Aldrich) for 30 min at 37 °C in the dark. The heart sections were washed twice with PBS and placed under an automatic fluorescence microscope (BX63, Olympus).

### Transmission electron microscopy (TEM)

2-mm × 2-mm × 2-mm pieces were removed from the apical region of freshly isolated mouse hearts and immediately fixed in 2.5% glutaraldehyde overnight. After embedding and cutting, they were observed under FEI Tecnai Spirit 120-kV TEM.

### Total iron ion content measurement

Hearts were isolated from mice, 0.1 g of fresh myocardium was taken from the septal region of freshly isolated hearts, and the iron ion content was determined using a total iron ion colorimetric test kit (Cat. E-BC-K772-M, Elabscience).

### Superoxide dismutase (SOD) activity level measurement

Hearts were isolated from mice, 0.1 g of fresh myocardial tissue was homogenized with magnetic beads at 4 °C and centrifuged at 4 °C, and the supernatants were removed. The level of SOD activity in the supernatant was assayed using a total SOD activity assay kit (Cat. S0109, Beyotime).

### Malondialdehyde (MDA) level measurement

Myocardial tissue was homogenized using precooled PBS, and the supernatant was centrifuged at 4 °C. The MDA content of the supernatant and plasma was determined using a malondialdehyde assay kit (Cat. S0131S, Beyotime).

### Western blot analysis

Tissues or cultured cells were lysed in RIPA buffer containing a protease inhibitor cocktail (Roche) for 30 min on ice. Protein (10–20 µg) was separated by SDS-PAGE. Western blotting was performed with NRF2 (1:2000, Cat. 16396-1-AP, Proteintech), ATF4 (1:500, Cat.10835-1-AP, Proteintech), SLC7A11 (1:2000, Car.26864-1-AP, Proteintech), GPX4 (1:1000, Cat. 67763-1-Ig, Proteintech) and GAPDH (1:10000, Cat. 60004-1-Ig, Proteintech) antibodies. The band intensities of target proteins were analyzed by using the ImageJ software.

### RT-qPCR

Mouse heart tissue was lysed using TRIzol reagent (Ambion), and total RNA was extracted. Total RNA was reverse transcribed using a reverse transcription kit (Bio-Rad). qPCR experiments were performed using a SYBR mixture (Bio-Rad). GAPDH was used to normalize and calculate fold ratios relative to the mRNA expression level of control samples. The PCR primer sequences used are shown in Additional file 1: Table S1.

### Statistical analysis

Data were statistically analyzed using GraphPad Prism 9.0 and then plotted on graphs and expressed as the mean ± SEM. The normality of the data was tested. Comparisons between two groups were performed using a two-tailed Student’s t-test with a 95% confidence interval. Comparisons for > 2 groups were analyzed using a one-way or two-way ANOVA followed by Bonferroni post hoc analysis. P < 0.05 was considered statistically significant.

## Results

### DEGs in HFpEF and functional enrichment analysis

**Fig. 1:**
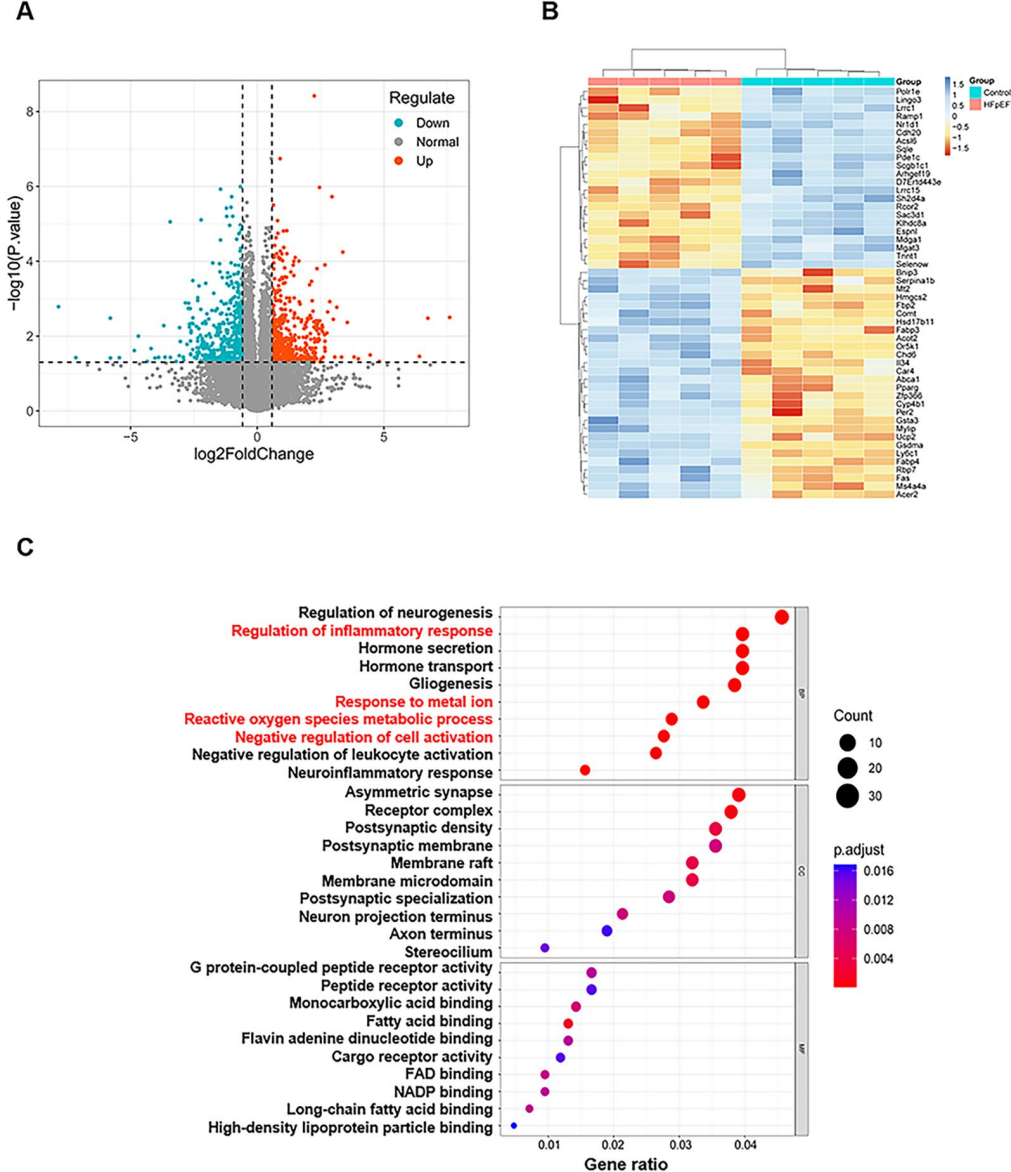
DEGs in HFpEF and results of GO and KEGG analysis.** A** Volcano plot of DEGs in GSE180065. **B** Clustered heatmap of the first 50 DEGs in GSE180065. **C** The enriched GO terms of DEGs in GSE180065

The microarray expression profiling dataset GSE180065 was obtained from the GEO database. Compared with controls, 952 differentially expressed genes were identified by differential analysis in HFpEF mouse heart samples, including 483 upregulated genes and 469 downregulated genes (Additional file 2: Table S2). A volcano plot was used to visualize the DEGs (Fig. 1A). The top 100 DEGs in the dataset were clustered and are shown in the form of a heatmap (Fig. 1B).

GO and KEGG analyses were used to further identify the major functions and pathways associated with the DEGs. The GO terms were categorized as biological processes (BP), cellular components (CC), and molecular functions (MF). For BP, four of the top ten GO terms are closely related to ferroptosis, including regulation of inflammatory response, response to metal ion, ROS metabolic process and negative regulation of cell activation. A chordal map was used to visualize the top five KEGG-enriched pathways, including the peroxisome proliferator-activated receptor (PPAR) signaling pathway, cholesterol metabolism, apelin signaling pathway, cytokine‒cytokine receptor interaction and Cushing syndrome (Additional file 3: Fig. S1A). KEGG enrichment analysis indicated that the DEGs are mainly involved in the PPAR signaling pathway. The PPAR signaling pathway is thought to be closely related to ferroptosis [17, 18]. Although the ferroptosis pathway was not directly enriched in the analysis of HFpEF mouse samples, both GO and KEGG results clearly demonstrated that the ferroptosis-related metabolic pathway was significantly enriched in the hearts of HFpEF mice. Specific GO and KEGG analysis results are shown in the supplementary material (Additional file 4: Table S3, Additional file 5: Table S4).

### Ferroptosis-related hub DEGs in HFpEF mice

**Fig. 2:**
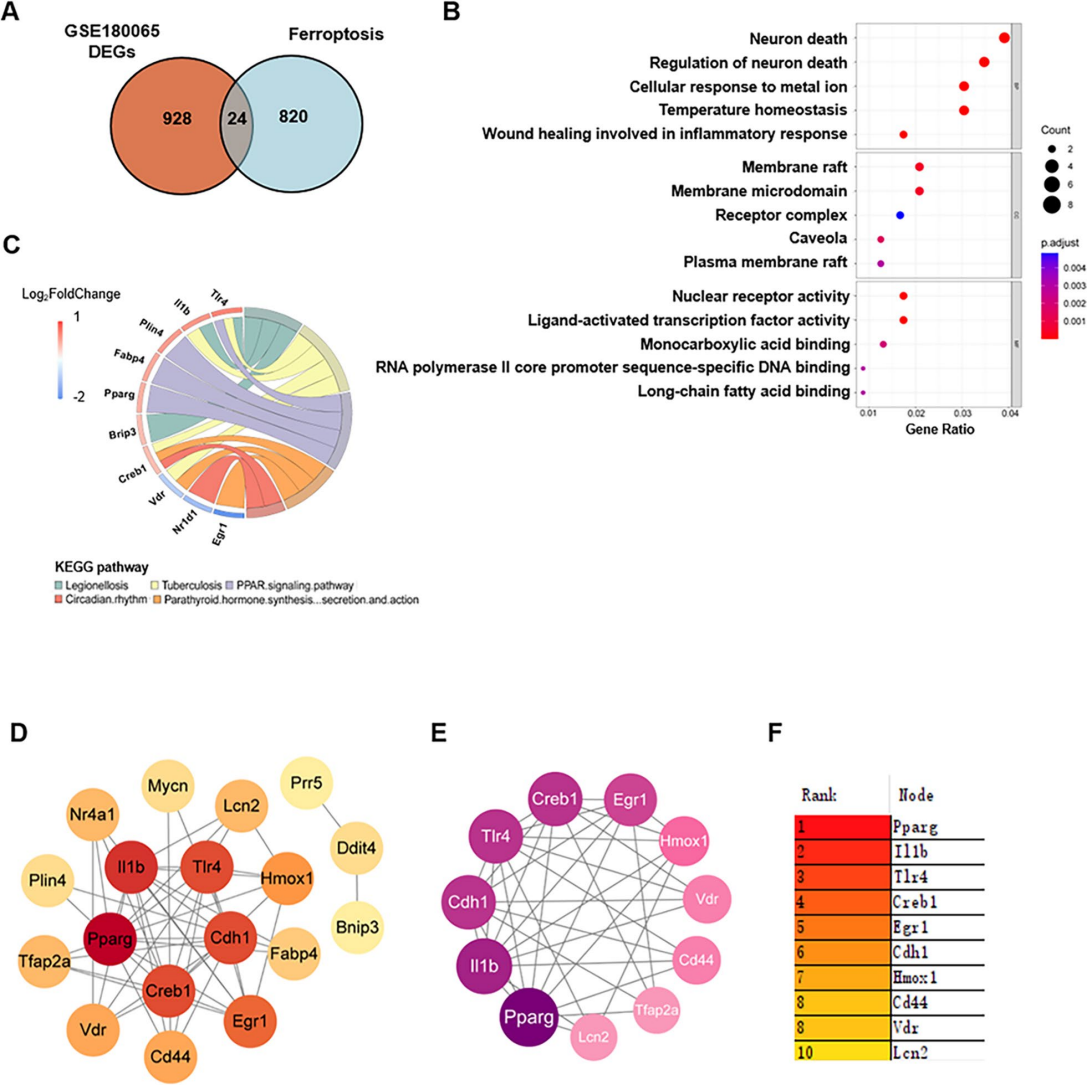
Identification of ferroptosis-related hub DEGs in HFpEF mice. **A** Venn plot of hub genes in the DEGs and FerrDb. **B** The enriched GO terms of ferroptosis-related DEGs. **C** KEGG pathway enrichment results for ferroptosis-related DEGs. **D** PPI network of ferroptosis-related DEGs. **E** A key cluster with 11 genes was further chosen as hub genes by MCODE. **F** Top 10 hub genes explored by CytoHubba

To further explore the potential mechanism of ferroptosis in HFpEF, we downloaded a dataset containing 844 genes from FerrDb and intersected them with DEGs in GSE180065 to identify ferroptosis-related genes. As shown by the Venn diagram, a total of 24 genes were obtained from the intersection of DEGs and ferroptosis-related genes, including 15 upregulated and 9 downregulated genes (Fig. 2A). DEGs in the intersection set were further categorized into ferroptosis drivers, ferroptosis suppressors, and unclassified via FerrDb (Table 1). We then analyzed GO and KEGG enrichment for these 24 ferroptosis-related DEGs and displayed the top five terms of BP, CC and MF as bubble plots and the top five KEGG enrichments as chord plots (Fig. 2B, C). All results of functional and pathway enrichment analyses on ferroptosis-related genes are shown in the supplementary material (Additional file 6: Table S5, Additional file 7: Table S6). In the BP part, functional analysis was mainly enriched in pathways such as neuron death and cellular response to metal ions. It is worth noting that the PPAR signaling pathway ranked in the top 5 in both KEGG analyses. Then, the PPI of the 24 ferroptosis-related DEGs was analyzed using the STRING online tool and visualized as a network by Cytoscape, including 18 nodes and 48 edges (Fig. 2D). Six genes were not related to other molecules. The MCODE plugin in Cytoscape was used to identify gene clusters in the ferroptosis hub DEGs. After filtering criteria were set to degree cutoff = 2, node score cutoff = 0.2, k hub = 2 and max depth = 100, a module with 11 nodes and 36 edges was identified with genes including *Lcn2*, *Hmox1*, *Il1b*, *Tlr4*, *Pparg*, *Egr1*, *Tfap2a*, *Creb1*, *Vdr*, *Cdh1*, and *Cd44* (Fig. 2E). We analyzed the PPI network again using the MCC algorithm in the plugin CytoHubba and screened 10 candidate hub DEGs, including *Cd44*, *Egr1*, *Cdh1*, *Pparg*, *Il1b*, *Tlr4*, *Hmox1*, *Vdr*, *Creb1*, and *Lcn2* (Fig. 2F). Combining these two results, we obtained 11 hub ferroptosis-related genes, including *Lcn2*, *Hmox1*, *Il1b*, *Tlr4*, *Pparg*, *Egr1*, *Tfap2a*, *Creb1*, *Vdr*, *Cdh1*, and *Cd44*. Seven of the upregulated genes included *Lcn2*, *Hmox1*, *Il1b*, *Pparg*, *Tlr4*, *Cd44*, and *Creb1*, and four downregulated genes included *Vdr*, *Cdh1*, *Egr1*, and *Tfap2a*.

**Table 1:** The ferroptosis-related DEGs were divided into ferroptosis driver, suppressor, and unclassified

Drive	Suppressor	Unclassified
Egr1, Hmox1, Il1β	Acot1, Cd44, Cdh1	Binp3, Ddit4, Hsd17b1
Pparg, Tlr4, Nr1d1	Fabp4, Lcn2, Nr4a1	Lurap1l, Plin4, Rgs4
Mycn, Ttpa	Tfap2α, Vdr, Creb1	
	Prr5	

### Validation of ferroptosis-related hub DEGs expression in HFpEF mice

**Fig. 3:**
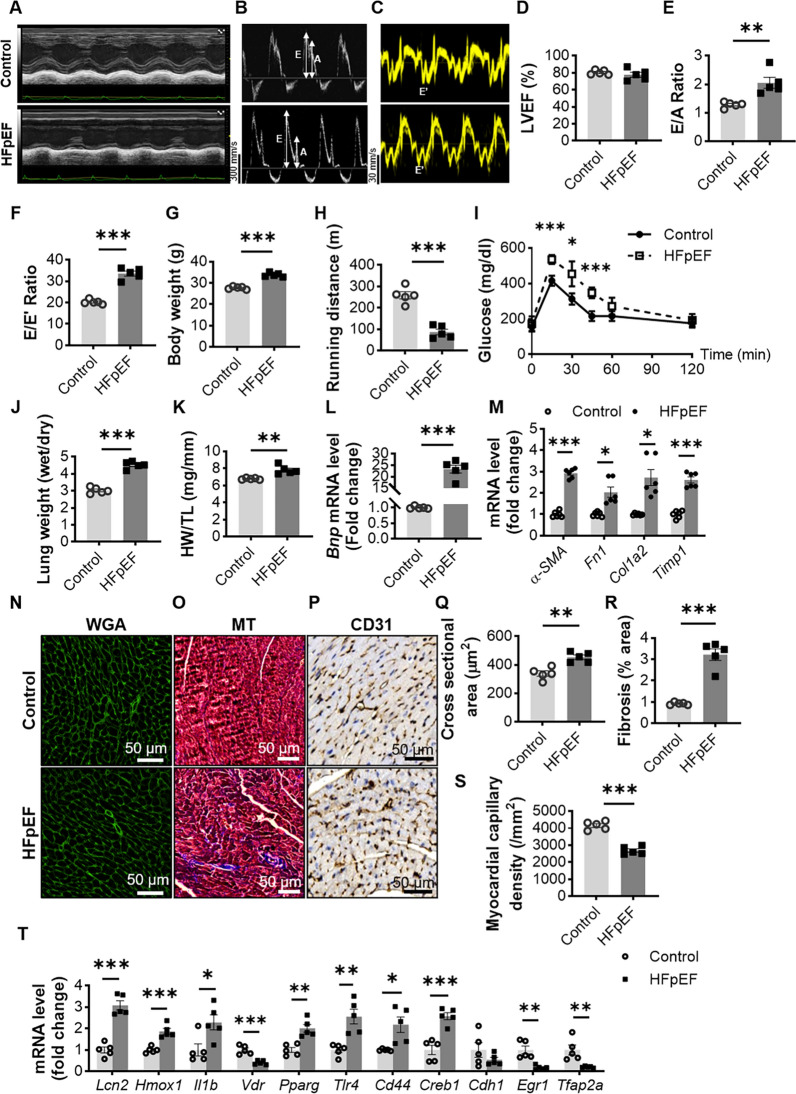
Validation of ferroptosis-associated hub DEGs expression in the hearts of HFpEF mice. **A**–**T** Control mice and HFpEF mice were both fed a normal diet (Control) or HFD+L-NAME (HFpEF) for 5 weeks. **A** Representative left ventricular M-mode echocardiographic tracings. **B** Percentage of LVEF, n = 5 mice per group. **C**, **D** Representative pulsed-wave and tissue Doppler tracings, n = 5 mice per group. **D**–**F** Percentage of ratio between mitral E wave and A wave; ratio between mitral E wave and e′ wave (E/e′); n = 5 mice per group. **G** Body weights of the two groups of mice after 5 weeks of rearing, n = 5 mice per group. **H** Running distances of the two groups of mice during the exercise tolerance test, n = 5 mice per group. **I** Mouse tail blood glucose at different time points in the intraperitoneal glucose tolerance test, n = 5 mice per group. **J** Ratio between wet and dry lung weight, n = 5 mice per group. **K** Ratio between heart weight and tibia length, n = 5 mice per group. **L**, **M** mRNA levels of *Bnp* and myocardial fibrosis-related genes (*α-SMA*, *Fn1*, *Col1a2*, *Timp1*) in myocardial tissues of mice in the two groups, n = 5 mice per group. **N**–**P** Representative images of WGA, MT and CD31-stained heart sections from control and HFpEF mice. **Q** WGA quantification of cardiomyocyte cross-sectional area, n = 5 mice per group (scale bar: 50 μm). **R** Percentage of fibrosis area in MT-stained heart sections, n = 5 mice per group (scale bar: 50 μm). **S** Myocardial capillary density, n = 5 mice per group (scale bar: 50 μm). **T** Ferroptosis-associated hub DEG mRNA expression in control and HFpEF mice. Statistical significance was calculated by Student’s t test; *P < 0.05, **P < 0.01, and ***P < 0.001

We first established a mouse model of HFpEF according to previous methods by inducing a distinct HFpEF phenotype in mice with HFD+L-NAME for 5 weeks [19]. The left ventricular ejection fraction (LVEF) remained unchanged in HFD− and L-NAME-fed mice compared with regular diet-fed mice (Fig. 3A, B). However, increased E/A ratio and E/e′ ratio indicate significant diastolic dysfunction in HFpEF mice. The increased left ventricular posterior wall thickness at diastole (LVPWd), left ventricular internal diameter at diastole (LVDd), left ventricular internal diameter at systole (LVDs) and interventricular septal end diastole (LVSd) also simultaneously demonstrated both increased stress loading and decreased diastolic function in HFpEF mice. (Fig. 3C–F and Additional file 8: Fig. S2A–D). HFD+L-NAME feeding also significantly induced weight gain, decreased exercise tolerance, and impaired glucose tolerance in mice (Fig. 3G–I and Additional file 8: Fig. S2E). We then found that HFpEF mice demonstrated significant cardiac enlargement and pulmonary congestion (Fig. 3J, K). The decline in cardiac function in HFpEF mice was also accompanied by a significant increase in the mRNA expression levels of *Bnp* and genes related to myocardial fibrosis, such as *α-SMA*, *Fn1*, *Col1a2*, and *Timp1* (Fig. 3L, M). At the same time, histologic findings also suggested that HFD+L-NAME led to cardiomyocyte enlargement, myocardial fibrosis, and a reduction in capillary density (Fig. 3N–S). To verify whether HFpEF mice showed overactivation of the immune system, we examined macrophage infiltration in the myocardium of HFpEF mice. The immunofluorescence results suggested significant macrophage recruitment in the myocardium of HFpEF mice (Additional file 8: Fig. S2F). Then, RT-qPCR was used to verify the transcript levels of previously screened genes associated with ferroptosis in the hearts of HFpEF mice. Compared to the control group, *Lcn2*, *Hmox1*, *Il1b*, *Pparg*, *Tlr4*, *Cd44*, and *Creb1* had significantly increased transcript levels in the HFpEF group (P < 0.05), while *Vdr*, *Egr1* and *Tfap2a* exhibited markedly decreased expression in the HFpEF group (P < 0.05). However, there was no significant difference in *Cdh1* expression between the HFpEF and control groups (Fig. 3T).

### Ferroptosis is activated in HFpEF

**Fig. 4:**
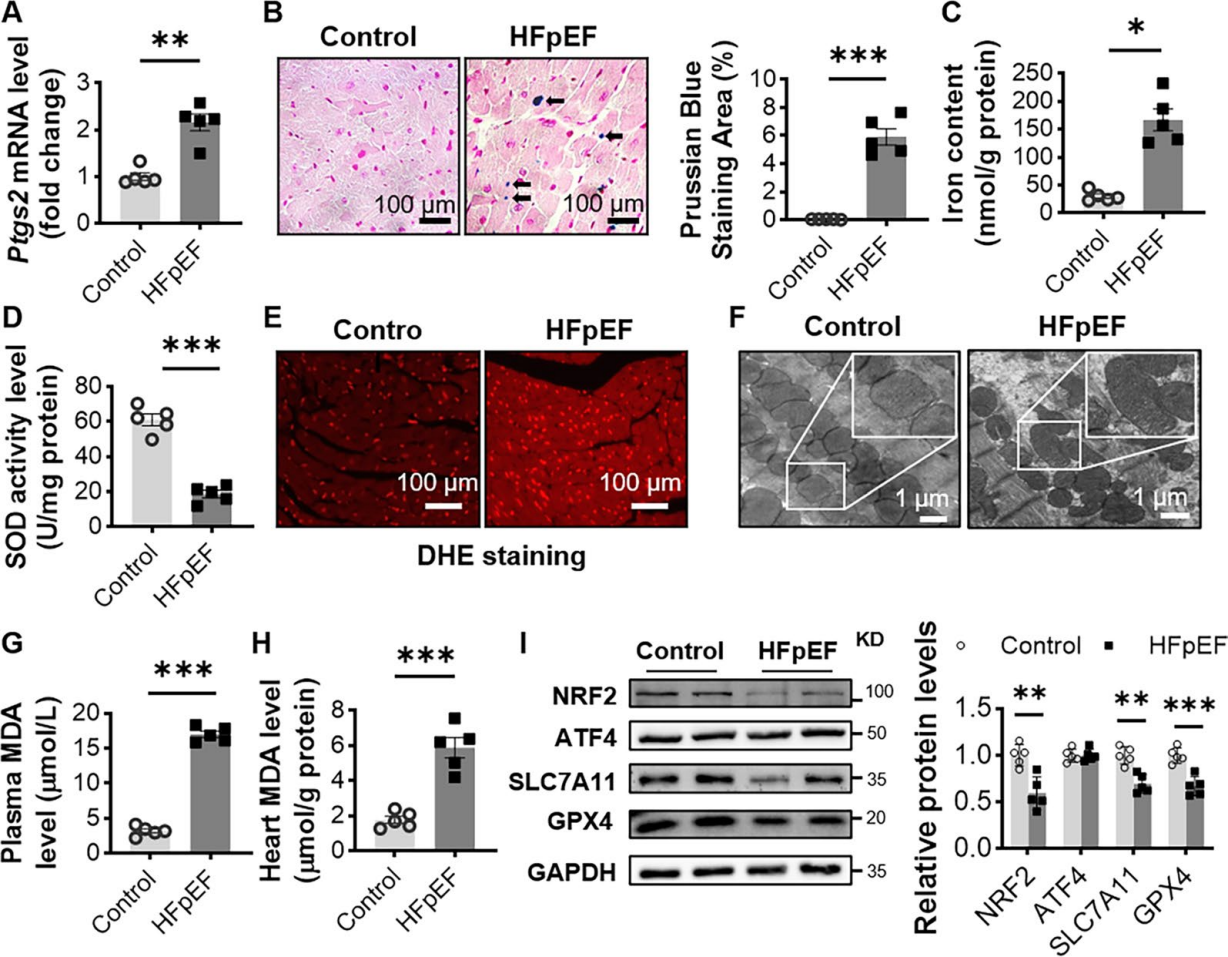
HFpEF mouse hearts showed abnormal activation of ferroptosis. **A** Relative expression of *Ptgs2* mRNA in myocardial tissues using RT-qPCR. *Gapdh* was used as an internal reference, n = 5 mice per group. **B** Representative Prussian blue-stained images of cross-sections of two groups of mouse hearts, and black arrows show blue-stained cardiomyocytes; Percentage of Prussian blue-stained positive cells, n = 5 mice per group. **C** Measurement of total iron ion content in myocardial tissue, n = 5 mice per group. **D** SOD activity level in myocardial tissue, n = 5 mice per group. **E** Representative DHE staining images of heart tissue, n = 5 mice per group. **F** Representative transmission electron microscopy images of heart tissue, n = 5 mice per group. **G**, **H** MDA levels in plasma and heart tissue, n = 5 mice per group. **I** Representative western blot images and average protein levels of SLC7A11, GPX4, NRF2 and ATF4 in control and HFpEF mouse hearts, n = 5 mice per group. Statistical significance was calculated by Student’s t test; *P < 0.05, **P < 0.01, and ***P < 0.001

Recent studies have shown that ferroptosis plays an important role in heart failure, while the pathogenesis of ferroptosis involved in HFpEF has not been elucidated [20, 21]. To determine the abnormal activation of ferroptosis in HFD+L-NAME-induced HFpEF mice and the potential mechanism behind it, we analyzed the mRNA expression of *Ptgs2*, a biochemical marker of ferroptosis [22]. As expected, *Ptgs2* mRNA was significantly upregulated more than in the hearts of HFpEF mice (Fig. 4A). Ferroptosis is mainly realized as intracellular iron overload, which further leads to lipid peroxidation [23]. We first stained the myocardial tissue with Prussian blue and measured the iron ion content in the myocardial tissue. Compared with the control group, we found that the percentage of trivalent iron-containing cells in the myocardial tissue of HFpEF mice was significantly increased and that the total iron content was significantly higher than that in control mice (Fig. 4B, C).

Excess iron promotes the production of mitochondrial ROS through the Fenton reaction, and mitochondrial ROS undergo lipid peroxidation due to the reaction of polyunsaturated fatty acids on the mitochondrial membrane [24, 25]. SOD is an enzyme that converts superoxide, such as ROS, into less damaging hydrogen peroxide. Compared with control mice, HFpEF mice showed an increase in ROS content in myocardial tissue and a significant decrease in SOD activity (Fig. 4D, E). Transmission electron microscopy was used to examine mitochondrial morphology. As expected, we found ultrastructural changes with ferroptosis features such as reduction in mitochondrial volume, increase in mitochondrial membrane density, and disappearance of mitochondrial cristaein the hearts of HFpEF mice (Fig. 4F). We further examined the level of MDA, a biomarker of lipid peroxidation and a critical indicator of ferroptosis in vivo, in the plasma and heart tissues of mice [23]. We found that MDA levels in plasma and myocardial tissue were significantly higher in HFpEF mice than in control mice (Fig. 4G, H). In addition to iron overload, loss of glutathione (GSH) is another key mechanism of ferroptosis, and intracellular GSH production requires SLC7A11, which transports cystine (a precursor of GSH) into the cytoplasm [26]. At the same time, inactivation of glutathione peroxidase 4 (Gpx4) also leads to loss of GSH and activation of ferroptosis [27]. We found that the protein levels of SLC7A11 and GPX4 in the myocardial tissue of HFpEF mice were significantly reduced (Fig. 4I). Nuclear factor red lineage 2-related factor 2 (NRF2) and activating transcription factor 4 (ATF4) can directly or indirectly regulate GPX4 activity [28, 29]. We then examined the protein expression levels of ATF4 and NRF2 in the myocardial tissues of the mice, and we found that there was no significant change in the levels of ATF4 in the myocardium of the HFpEF mice, but NRF2 appeared to be significantly reduced (Fig. 4I). These results suggest that HFD and circulating high load (L-NAME-induced) may regulate GPX4 activity through NRF2 and thus cause ferroptosis.

### Ferrostatin-1 inhibits ferroptosis and alters the transcript levels of ferroptosis hub DEGs

**Fig. 5:**
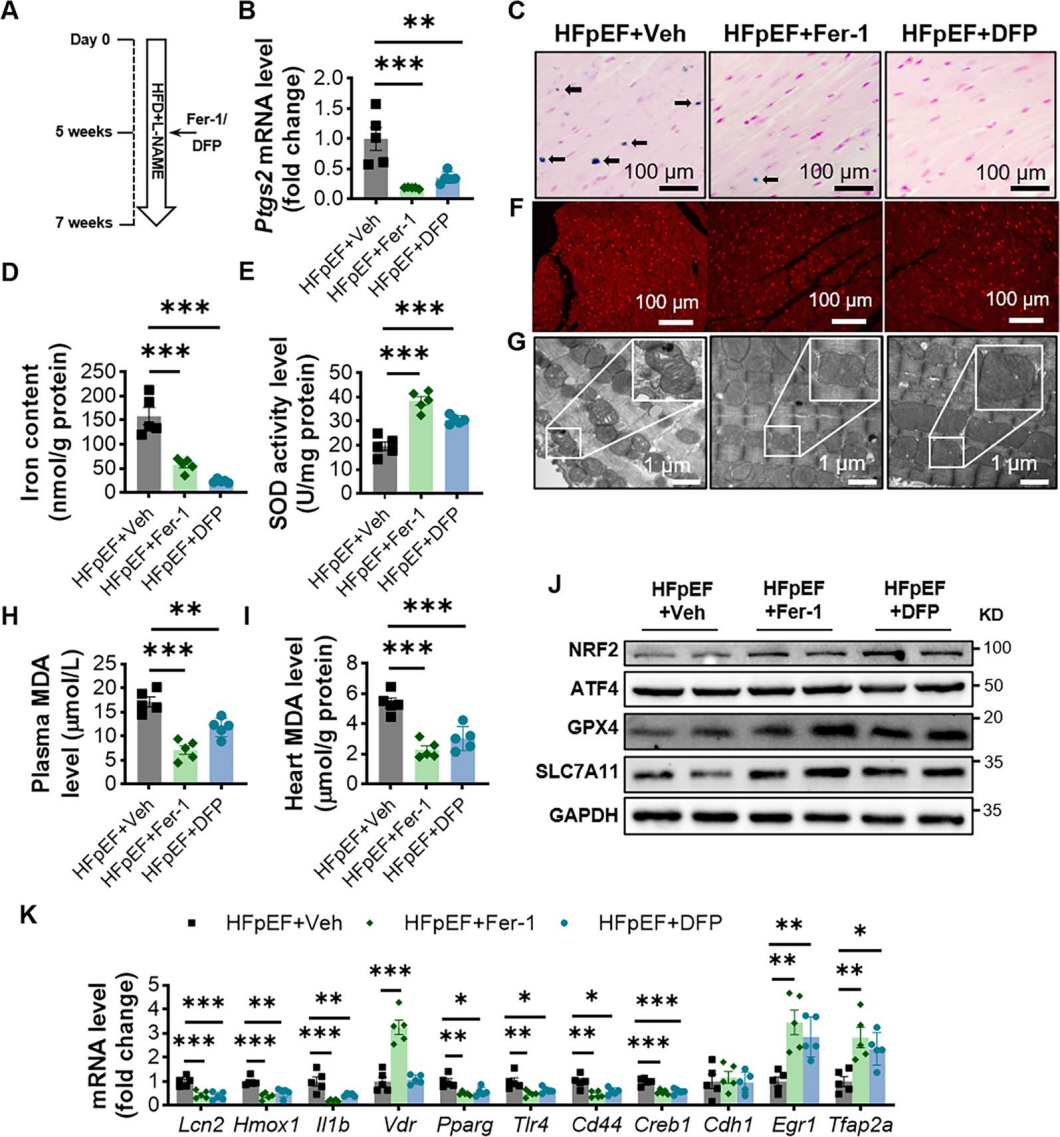
Ferrostatin-1 inhibits ferroptosis and alters the expression levels of ferroptosis hub DEGs. **A** Experimental design. **B** Relative expression of *Ptgs2* mRNA in myocardial tissues using RT-qPCR. *Gapdh* was used as an internal reference, n = 5 mice per group. **C** Representative Prussian blue-stained images of cross-sections of two groups of mouse hearts, and black arrows show blue-stained cardiomyocytes; Percentage of Prussian blue-stained positive cells, n = 5 mice per group. **D** Measurement of total iron ion content in myocardial tissue, n = 5 mice per group. **E** SOD activity level in myocardial tissue, n = 5 mice per group. **F** Representative DHE staining images of heart tissue, n = 5 mice per group. **G** Representative transmission electron microscopy images of cardiac tissue, n = 5 mice per group. **H**, **I** MDA levels in plasma and heart tissue, n = 5 mice per group. **J** Representative western blot images and average protein levels of SLC7A11, GPX4, NRF2 and ATF4 in HFpEF+Veh, HFpEF+Fer-1 and HFpEF+DFP mouse hearts, n = 5 mice per group. **K** Ferroptosis-associated hub DEGs mRNA expression in HFpEF+Veh and HFpEF+Fer-1 mice. Statistical significance was calculated by one-way or two-way ANOVA; *P < 0.05, **P < 0.01, and ***P < 0.001

To investigate whether ferroptosis is involved in the development of HFpEF, we used two ferroptosis inhibitors, Ferrostatin-1 (Fer-1) and deferiprone (DFP). Fer-1 directly inhibits lipid peroxidation, whereas DFP acts as an iron specific chelator and is used in the clinical treatment of iron overload diseases [28, 30]. We divided HFD+L-NAME-fed mice for 5 weeks into three groups, one receiving intraperitoneal Fer-1 for 2 weeks, one receiving intraperitoneal DFP for 2 weeks and the other receiving intraperitoneal injection of vehicle for 2 weeks (Fig. 5A). First, we found that the *Ptgs2* mRNA expression levels were significantly reduced in the myocardial tissue of the HFpEF+Fer-1 and HFpEF+DFP group (Fig. 5B). Meanwhile, the number of trivalent iron-containing cells in the myocardial tissue of mice was significantly reduced, and the total iron content were also significantly lower after Fer-1 or DFP injection (Fig. 5C, D and Additional file 9: Fig. S3A). For HFpEF mice injected intraperitoneally with Fer-1 or DFP, a significant increase in myocardial tissue SOD activity was accompanied by a significant decrease in ROS (Fig. 5E, F). Transmission electron microscopy results also suggested that Fer-1 and DFP prevented mitochondrial deformation caused by HFD+L-NAME (Fig. 5G). We also found a significant decrease in MDA levels in the heart and blood after intraperitoneal injection of Fer-1 or DFP (Fig. 5H, I). The western blot results suggested that compared with HFpEF+Veh mice, the myocardial tissues of HFpEF+Fer-1 and HFpEF+DFP mice showed a significant increase in the levels of SLC7A11 and GPX4 protein expression, as well as a significant increase in the expression of NRF2, which directly regulates the activity of GPX4, but there was no difference in the content of ATF4 (Fig. 5J and Additional file 9: Fig. S3B). Together, these results suggest that Fer-1 and DFP inhibit ferroptosis in the hearts of HFpEF mice.

Then, we examined the expression levels of ferroptosis hub DEGs after intraperitoneal injection of Fer-1 or DFP. The results indicated that the transcript levels of *Lcn2*, *Hmox1*, *Il1b*, *Pparg*, *Tlr4*, *Cd44* and *Creb1* in the hearts of mice in the Fer-1 or DFP group were significantly lower than those in the vehicle group, and the transcript levels of *Egr1* and *Tfap2a* were significantly higher than those in the HFpEF+Veh group (Fig. 5K). For HFpEF mice, intraperitoneal injection of Fer-1 but not DFP increases *Vdr* expression levels in myocardial tissue. However, we did not find significant differences in *Cdh1* mRNA expression among the three groups of mice (Fig. 5K).

### The HFpEF phenotype is ameliorated by inhibition of ferroptosis

**Fig. 6:**
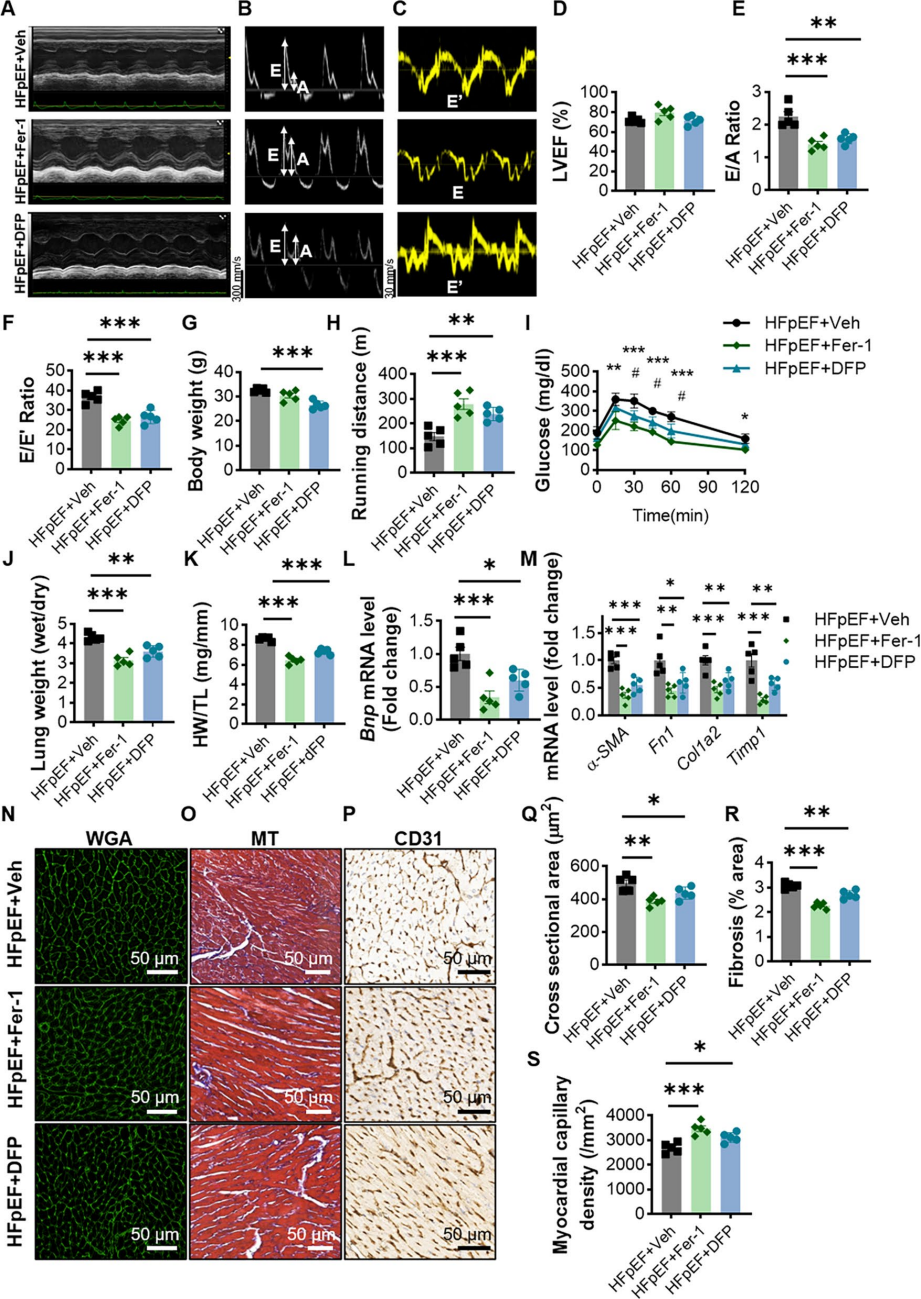
Inhibition of ferroptosis alleviates HFpEF. **A** Representative left ventricular M-mode echocardiographic tracings. **B** Representative pulsed-wave Doppler tracings. **C** Representative tissue Doppler tracings. **D**–**F** Percentage of LVEF; ratio between mitral E wave and A wave; ratio between mitral E wave and e′ wave (E/e′); n = 5 mice per group. **G** Body weights of the two groups of mice after 7 weeks of rearing, n = 5 mice per group. **H** Running distances of the two groups of mice during the exercise tolerance test, n = 5 mice per group. **I** Mouse tail blood glucose at different time points in the intraperitoneal glucose tolerance test, n = 5 mice per group. *Indicates the comparison between the HFpEF+Veh and HFpEF+Fer-1 group; ^#^indicates the comparison between the HFpEF+Veh and HFpEF+DFP group. **J** Ratio between wet and dry lung weight, n = 5 mice per group. **K** Ratio between heart weight and tibia length, n = 5 mice per group. **L**, **M** mRNA levels of *Bnp* and myocardial fibrosis-related genes (*α-SMA*, *Fn1*, *Col1a2*, *Timp1*) in myocardial tissues of mice in the two groups, n = 5 mice per group. **N**–**P** Representative images of WGA, MT and CD31-stained heart sections from the two groups of mice. **Q** WGA quantification of cardiomyocyte cross-sectional area, n = 5 mice per group (scale bar: 50 μm). **R** Percentage of fibrosis area in MT-stained heart sections, n = 5 mice per group (scale bar: 50 μm). **S** Myocardial capillary density, n = 5 mice per group (scale bar: 50 μm). Statistical significance was calculated by one-way or two-way ANOVA; *P < 0.05, **P < 0.01, and ***P < 0.001

We further examined the HFpEF-related phenotypes in three groups of mice to determine whether attenuating ferroptosis could mitigate the progression of HFpEF. As shown in the echocardiograms, although the left ventricular ejection fraction did not change much in either group, the E/A ratio, E/e′ ratio, LVPWd, LVDd, LVDs and LVSd of the mice in the HFpEF+Fer-1 and HFpEF+DFP groups were significantly lower than those in the HFpEF+Veh group, indicating that the diastolic function of the heart was significantly improved (Fig. 6A–F and Additional file 9: Fig. S3C–F). Intraperitoneal injection of Fer-1 did not significantly reduce HFpEF body weight, but DFP did reduce body weight in mice (Fig. 6G). Exercise tolerance and glucose tolerance were significantly higher in the HFpEF+Fer-1 group than in the HFpEF+Veh group, but there was no significant difference in the HFpEF+DFP group (Fig. 6H, I and Additional file 8: Fig. S2G). Meanwhile, inhibition of ferroptosis in HFpEF mice demonstrated robust reductions in heart size and lung weight compared with those in the HFpEF+Veh group, suggesting significant remission of pulmonary congestion and preclinical heart failure states (Fig. 6J, K). The RT-qPCR results indicated that the transcript levels of myocardial fibrosis-related genes and *Bnp* were significantly reduced in the Fer-1 or DFP treatment group (Fig. 6L, M). The HFpEF+Veh group showed marked cardiomyocyte hypertrophy, cardiac fibrosis and reduced myocardial capillary density, which were markedly ameliorated by inhibition of ferroptosis (Fig. 6N–S). Fer-1 and DFP also significantly reduced macrophage infiltration within the myocardial tissue of HFpEF mice (Additional file 9: Fig. S3H). The above results prove that inhibition of abnormally activated ferroptosis in HFpEF mice significantly ameliorated HFpEF-related phenotypes such as diastolic dysfunction of the heart and pulmonary congestion in HFpEF mice.

## Discussion

Our study uncovered for the first time the key genes associated with ferroptosis in HFpEF disease, and based on this, we further explored the potential mechanism of action of ferroptosis in HFpEF. We obtained 24 DEGs, including 15 upregulated genes and 9 downregulated genes, by analyzing the GSE180065 dataset downloaded from the NCBI database and intersecting the DEGs with the gene set downloaded from FerrDB. The STRING online tool and the MCODE and CytoHubba plugins in Cytoscape were then used to screen 11 ferroptosis-related hub DEGs. Abnormal activation of ferroptosis was found in mice constructed with the HFpEF phenotype using the ‘two hit’ model, and changes in the expression level of ferroptosis-associated hub DEGs were determined using RT-qPCR. After intraperitoneal injection of Ferrostatin-1, the expression of ferroptosis-related core genes in the hearts of HFpEF mice was identified, while results such as cardiac ultrasound and pathology suggested that inhibition of the ferroptosis pathway could significantly improve the HFpEF phenotype.

Previous studies have shown that HFpEF, as a distinct and prevalent form of heart failure, is significantly associated with metabolic syndrome. Increased visceral fat due to obesity, dyslipidemia, type 2 diabetes mellitus and hypertension associated with a high-salt diet serves as an important risk factor for HFpEF [31]. From another perspective, the major role played by visceral adipose tissue in the development of HFpEF also suggests that the pathogenesis of HFpEF is inextricably linked to systemic metabolic disturbances [32, 33]. Metabolic syndrome puts the body in a chronic inflammatory state for a long time, and the imbalance of oxidative stress and antioxidants in the body further causes abnormal accumulation of ROS, microvascular injury, compensatory hypertrophy of cardiomyocytes and myocardial fibrosis, and finally develops into diastolic dysfunction and even HFrEF [34]. Meanwhile, the pathogenesis of ferroptosis in heart failure, which is mainly characterized by iron overload and lipid peroxidation, has been gradually elucidated, suggesting the potential pathogenesis of HFpEF [25, 35]. Previous studies have shown that when ferroptosis is increased, the immune system is reactively activated and involved in regulating the immune response [36–38]. Patients with HFpEF exhibit higher levels of inflammation and increased inflammatory factors in the somatic circulation, leading to endothelial cell injury [39, 40]. Damaged endothelial cells accumulate in the microcirculation through endothelial cell adhesion molecules and e-selectin, which recruit immune cells, especially macrophages, to the microcirculation, further leading to microcirculatory blockage, and inflammatory factors secreted by immune cells, such as Il-1b, Il-6, and TNF-a, in turn activate the inflammatory system, leading to a vicious cycle [41–44].

However, no study has examined the independent role of ferroptosis in HFpEF. Therefore, in this study, we attempted to explore the association between ferroptosis and HFpEF through bioinformatics analysis combined with experimental validation. The results obtained from our functional and pathway enrichment analysis of 952 DEGs suggest a major enrichment in immunoinflammatory pathways, such as regulation of inflammatory response, response to metal ion, ROS metabolic process, PPAR signaling pathway, negative regulation of cell activation and negative regulation of leukocyte activation, suggesting that the development of HFpEF is indeed related to immune and inflammatory interactions. Then, a total of 24 DEGs overlapping in ferroptosis and HFpEF were identified in this study. We analyzed the obtained ferroptosis-related DEGs for functional and pathway enrichment. Interestingly, both enrichment analyses enriched the PPAR signaling pathway, monocarboxylic acid binding and long-chain fatty acid binding. The PPAR signaling pathway is closely related to fatty acid metabolism and immunoregulation [45]. The involvement of the PPAR signaling pathway in HFpEF does not appear to have been fully elucidated. A report found that SIRT6 in rat vascular endothelial cells improves cardiac diastolic function by inhibiting PPARγ expression in endothelial cells [46], while another article suggested that PPARγ activation may reduce systolic blood pressure and left ventricular mass and attenuate myocardial fibrosis in HFpEF rats by inhibiting the Wnt-β-catenin pathway [47]. All of this evidence suggests that activation of the PPAR signaling pathway plays a role in the development of HFpEF, but further experimental validation is still needed.

Then, we screened ferroptosis-related DEGs in GSE180065 by MCODE and CytoHubba plugins in Cytoscape and obtained 11 hub DEGs. *Pparg*, the top-ranked hub DEGs by CytoHubba, encodes the PPAR receptor γ subtype [48]. *Pparg* is predominantly expressed in adipose tissue and plays a key role in lipid metabolism and the maintenance of energy homeostasis [48, 49]. For the heart, an organ that primarily utilizes fatty acids, *Pparg* deficiency leads to ventricular contractile dysfunction and intracellular fatty acid overload in cardiomyocytes in mice, whereas pioglitazone, an agonist of *Pparg*, reverses hypoxia-induced pulmonary hypertension and ventricular remodeling in rats [50]. Similarly, our results also confirmed that the transcriptional level of *Pparg* was significantly upregulated in the hearts of HFpEF mice and that the ferroptosis inhibitor Ferrostatin-1 downregulated its expression. *Il1b* encodes a protein that is a cytokine produced primarily by activated macrophages and is involved in the immune response and assembly of inflammatory vesicles such as NLRP3 [51]. Studies have shown that the assembly of NLRP3 inflammatory vesicles can further cause ferroptosis [52]. In addition, NLRP3 inflammatory vesicles have been shown to be closely associated with cardiac inflammation and myocardial hypertrophy [43, 53]. *Tlr4* encodes Toll-like receptor 4 protein, which is involved in immune responses such as pathogen recognition and immune activation. Toll-like receptor 4 activation by LPS can activate the NF-κB pathway, which leads to increased HIF-1a expression, further causing ferroptosis [54]. Meanwhile, the NF-κB pathway and the JAK/STAT3 pathway, in which Toll-like receptor 4 is involved, are thought to be involved in cardiac hypertrophy [55]. *Creb1* encodes cAMP responsive element binding protein 1, a member of the leucine zipper family of cAMP-responsive element binding proteins. A study showed that *Creb1* can inhibit ferroptosis by binding to the GPX4 promoter region and increasing GPX4 activity to inhibit lipid peroxidation [56]. In addition, a study suggested that the protein expression of CREB is significantly reduced in the hearts of patients with heart failure, similar to what we observed in HFpEF mice [57]. *Egr1* is mainly involved in the regulation of gene expression and hormone synthesis processes [58]. In the heart, *Egr1* has been shown to be associated with myocardial growth [59]. We observed downregulation of EGR expression in HFpEF mice, which was reversed after the use of a ferroptosis inhibitor. However, one report suggests that *Egr1* is significantly associated with right ventricular hypertrophy due to pulmonary hypertension [60]. The role of *Egr1* in the development of HFpEF still needs further validation. *Hmox1* encodes heme oxygenase, a key rate-limiting enzyme that catalyzes the production of iron ions from heme. Unlike other screened hub DEGs, Hmox1 has been more thoroughly investigated to further contribute to myocardial hypertrophy and myocardial injury through activation of ferroptosis [9, 61]. However, the role of *Hmox1* in HFpEF remains unresolved. *Cd44* is a single transmembrane glycoprotein involved in several biological processes [62]. *Cd44* induces inflammation in the body circulation, myocardial fibrosis and cardiac hypertrophy through binding to its ligand hyaluronic acid [63]. *Vdr* encodes the vitamin D receptor. A study showed that myocardial hypertrophy occurs in *Vdr* knockout mice due to activation of the angiotensin system [64]. However, a previous study suggested that activation of zebrafish cardiac *Vdr* leads to increased proliferation and regeneration of the heart [65]. *Lcn2* encodes lipocalcin 2 and can reduce inflammation by binding to iron carriers [66]. Several studies suggest that LCN2 protein is a risk factor for atherosclerosis and myocardial ischemic injury [67–69]. A recent study showed that the protein level of LCN2 in the heart is strongly associated with nonphysiological enlargement of the heart [70]. This is consistent with our observation of increased Lcn2 expression in the hearts of HFpEF mice, but whether *Lcn2* is involved in HFpEF through ferroptosis requires further verification. *Tfap2a* encodes a transcription factor, activator protein 2 (AP-2), which is primarily associated with the regulation of gene transcription [71]. Currently, there are still too few studies on *Tfap2a* in cardiac diseases. FU Muller et al. found that AP-2 expression was increased in the hearts of patients with idiopathic dilated cardiomyopathy and that AP-2 was associated with cardiomyocyte apoptosis in rats [72]. This is different from our finding of reduced *Tfap2a* transcript levels in HFpEF mice, and we speculate that this may be due to the large differences in pathophysiologic mechanisms between HFpEF and idiopathic dilated cardiomyopathy.

We have also experimentally demonstrated that aberrant activation of ferroptosis does exist in the hearts of HFpEF mice and have found a remission of the HFpEF phenotype and altered expression levels of pivotal genes after ferroptosis is inhibited. One thing to point out is that in the animal experiments section, our HFpEF mouse model remained consistent with the dataset, and both were fed a high-fat diet and L+NAME for 5 weeks. However, in the HFpEF+Fer-1, HFpEF+DFP and HFpEF+Veh groups, we took into account the normal clinical course of treatment, i.e., treatment is usually administered after diagnosis, so we continued to feed a high-fat diet and L-NAME while intraperitoneal injection of Fer-1 or DFP was given for 2 weeks after successful HFpEF modeling.

It is important to note that the mechanism of HFpEF occurrence has been shown to be significantly related to lipid metabolism [34]. A recent human plasma and endomyocardial metabolomics study reported that multiple metabolites of fatty acid oxidation was markedly lower in the myocardium of HFpEF patients, and the expression of genes related to fatty acid metabolism was also reduced in the HFpEF group [73]. In another study, accumulation of epicardial adipose tissue was observed, which was associated with worse hemodynamic and metabolic profiles, as well as survival, in HFpEF patients [74]. Lipid accumulation induces cardiac lipotoxicity and subsequent myocardial dysfunction through multiple pathways, including increasing the generation of ROS [75].

Nevertheless, this study still has some limitations. First, our study is based on a public dataset, and the results obtained are largely exploratory and may differ somewhat from the actual results. At the same time, the dataset we downloaded from FerroDb does not summarize all ferroptosis-related genes and may contain some degree of omission. Second, due to the small number of studies on HFpEF, we only obtained a dataset from the transcriptome analysis of cardiac tissues from HFpEF mice, and the results obtained from the analysis may not be well represented. More regrettably, we did not obtain and analyze a transcriptome dataset from humans. We were also unable to obtain cardiac tissues from patients for further validation because of experimental ethical reasons. Although human genes and mouse genes share a certain degree of homology, there are still major differences in structure and function. HFpEF as a systemic inflammatory disease, we have not explored the role that other tissues such as liver and skeletal muscle play in the progression of HFpEF and the contribution of inflammation and immunity in HFpEF. It is worth noting that the specific metabolic mechanisms involved in the involvement of ferroptosis in the development of HFpEF were not further explored in our study, and whether these metabolic alterations regulate ferroptosis and HFpEF warrants a more comprehensive study in the future.

## Conclusion

In conclusion, we obtained common hub DEGs associated with ferroptosis and HFpEF by bioinformatics analysis. We also verified the corresponding changes in the hub DEGs by RT-qPCR in the hearts of HFpEF mice and in the hearts after the use of ferroptosis inhibitors, suggesting that these hub DEGs may be involved in the development of HFpEF through ferroptosis. Eleven hub DEGs could be potential targets of drug action for the treatment of HFpEF, but further detailed experimental validation is still needed.

### Supplementary Information

**Additional file 1: Table S1.** Primer sequences for RT qPCR.

** Additional file 2: Table S2.** Differentially expressed genes involved in HFpEF samples.

**Additional file 3: Figure S1.** Top five KEGG pathway enrichment results in GSE180065.

** Additional file 4: Table S3.** GO enrichment analysis of DEGs from GSE180065.

** Additional file 5: Table S4.** KEGG pathway enrichment analysis of DEGs from GSE180065.

**Additional file 6: Table S5.** GO enrichment analysis of ferroptosis-related DEGs.

** Additional file 7: Table S6.** KEGG pathway enrichment analysis of ferroptosis-related DEGs.

**Additional file 8: Figure S2.** Additional material related to Figure 3. **A** LVPWd, n = 5 mice per group. **B** LVDd, n = 5 mice per group. **C** LVDs, n = 5 mice per group. **D** LVSd, n = 5 mice per group. **E** Area under the curve of the intraperitoneal glucose tolerance test experiment, n = 5 mice per group. **F** Representative images of immunohistochemical staining showing macrophage infiltration in the heart tissue, n = 5 mice per group. Statistical significance was calculated by Student’s t test; *P < 0.05, **P < 0.01, ***P < 0.001.

**Additional file 9: Figure S3.** Fer-1 and DFP inhibit intramyocardial ferroptosis and ameliorate the HFpEF phenotype. **A** Percentage of Prussian blue-stained positive cells, n = 5 mice per group. **B** Average protein levels of SLC7A11, GPX4, NRF2 and ATF4 in mouse heart tissue, n = 5 mice per group. **C** LVPWd, n = 5 mice per group. **D** LVDd, n = 5 mice per group. **E** LVDs, n = 5 mice per group. **F** LVSd, n = 5 mice per group. **(G)** Area under the curve of the intraperitoneal glucose tolerance test experiment, n = 5 mice per group. **H** Representative images of immunohistochemical staining showing macrophage infiltration in the heart tissue, n = 5 mice per group. Statistical significance was calculated by one-way ANOVA; *P < 0.05, **P < 0.01, ***P < 0.001.

## Data Availability

The datasets analyzed during the current study are available in the GEO database (https://www.ncbi.nlm.nih.gov/geo/) and FerrDb (http://www.zhounan.org/ferrdb/).

## Abbreviations

ATF4: Activating transcription factor 4
BP: Biological process
CC: Cellular component
DEGs: Differentially expressed genes
DFP: Deferiprone
DHE: Dihydroethidium
FerrDb: Ferroptosis database
Fer-1: Ferrostatin-1
GO: Gene ontology
GPX4: Glutathione peroxidase 4
GSH: Glutathione
HFD: High-fat diet
HFpEF: Heart failure with preserved ejection fraction
HFrEF: Heart failure with reduced ejection fraction
KEGG: Kyoto Encyclopedia of Genes and Genomes
L-NAME: *N*^ω^-Nitro-l-arginine methyl ester
LVDd: Left ventricular internal diameter at diastole
LVDs: Left ventricular internal diameter at systole
LVPWd: Left ventricular posterior wall thickness at diastole
LVSd: Interventricular septal end diastole
MDA: Malondialdehyde
MF: Molecular function
MT: Masson’s trichrome
NCBI: National Center for Biotechnology Information
NRF2: Nuclear factor red lineage 2-related factor 2
PPAR: Peroxisome proliferator-activated receptors
PPI: Protein‒protein interaction
ROS: Reactive oxygen species
RT-qPCR: Real-time quantitative polymerase chain reaction
SOD: Superoxide dismutase
WGA: Wheat germ agglutinin
