# Supplementary material for: Inhibition of ferroptosis reverses heart failure with preserved ejection fraction in mice
Source: J Transl Med. 2024 Feb 24;22:199. doi: 10.1186/s12967-023-04734-y (PMC10894491; doi:10.1186/s12967-023-04734-y)
Supplement: Supplementary file 1 — Additional file 1: Table S1. Primer sequences for RT qPCR. [file 12967_2023_4734_MOESM1_ESM.docx]

Table S1. Primer Sequence for RT qPCR.

| Gene (Mouse) | Forward | Reverse |
| --- | --- | --- |
| *Gapdh* | AGGTCGGTGTGAACGGATTTG | TGTAGACCATGTAGTTGAGGTCA |
| *Bnp* | ACAATCCACGATGCAGAAGCT | GGGCCTTGGTCCTTTGAGA |
| *Il1β* | GCAACTGTTCCTGAACTCAACT | ATCTTTTGGGGTCCGTCAACT |
| *Lcn2* | TGGCCCTGAGTGTCATGTG | CTCTTGTAGCTCATAGATGGTGC |
| *Cd44* | AGCAGCGGCTCCACCATCGAGA | TCGGATCCATGAGTCACAGTG |
| *Cdh1* | TCGGAAGACTCCCGATTCAAA | CGGACGAGGAAACTGGTCTC |
| *Pparg* | GGAAGACCACTCGCATTCCTT | GTAATCAGCAACCATTGGGTCA |
| *Creb1* | AGCAGCTCATGCAACATCATC | AGTCCTTACAGGAAGACTGAACT |
| *Hmox1* | CACTTCGTCAGAGGCCTGCTA | GTCTGGGATGAGCTAGTGCTGAT |
| *Tfap2α* | GAAGACTGCGAGGACCGTC | GAAGTCGGCATTAGGGGTGTG |
| *Tlr4* | ATGGCATGGCTTACACCACC | GAGGCCAATTTTGTCTCCACA |
| *Egr1* | TCGGCTCCTTTCCTCACTCA | CTCATAGGGTTGTTCGCTCGG |
| *Vdr* | ACCCTGGTGACTTTGACCG | GGCAATCTCCATTGAAGGGG |
| *Ptgs2* | GCGACATACTCAAGCAGGAGCA | AGTGGTAACCGCTCAGGTGTTG |
| *α-SMA* | AGAACACGGCATCATCACCAACTG | AGTCACACCATCTCCAGAGTCCAG |
| *Col1A2* | GCTTGCAACACAAAGGTGCT | GTGGCCTGCATTCCTGTAGT |
| *Fn1* | GCAAGCCTGAGCCTGAAGAGAC | CAGTCCACCATCATCCAGCCTTG |
| *Timp1* | GCAGATATCCGGTACGCCTACAC | AAGTGACGGCTCTGGTAGTCCTC |
