# Supplementary figures and images for: Inhibition of ferroptosis reverses heart failure with preserved ejection fraction in mice
Source: J Transl Med. 2024 Feb 24;22:199. doi: 10.1186/s12967-023-04734-y (PMC10894491; doi:10.1186/s12967-023-04734-y)

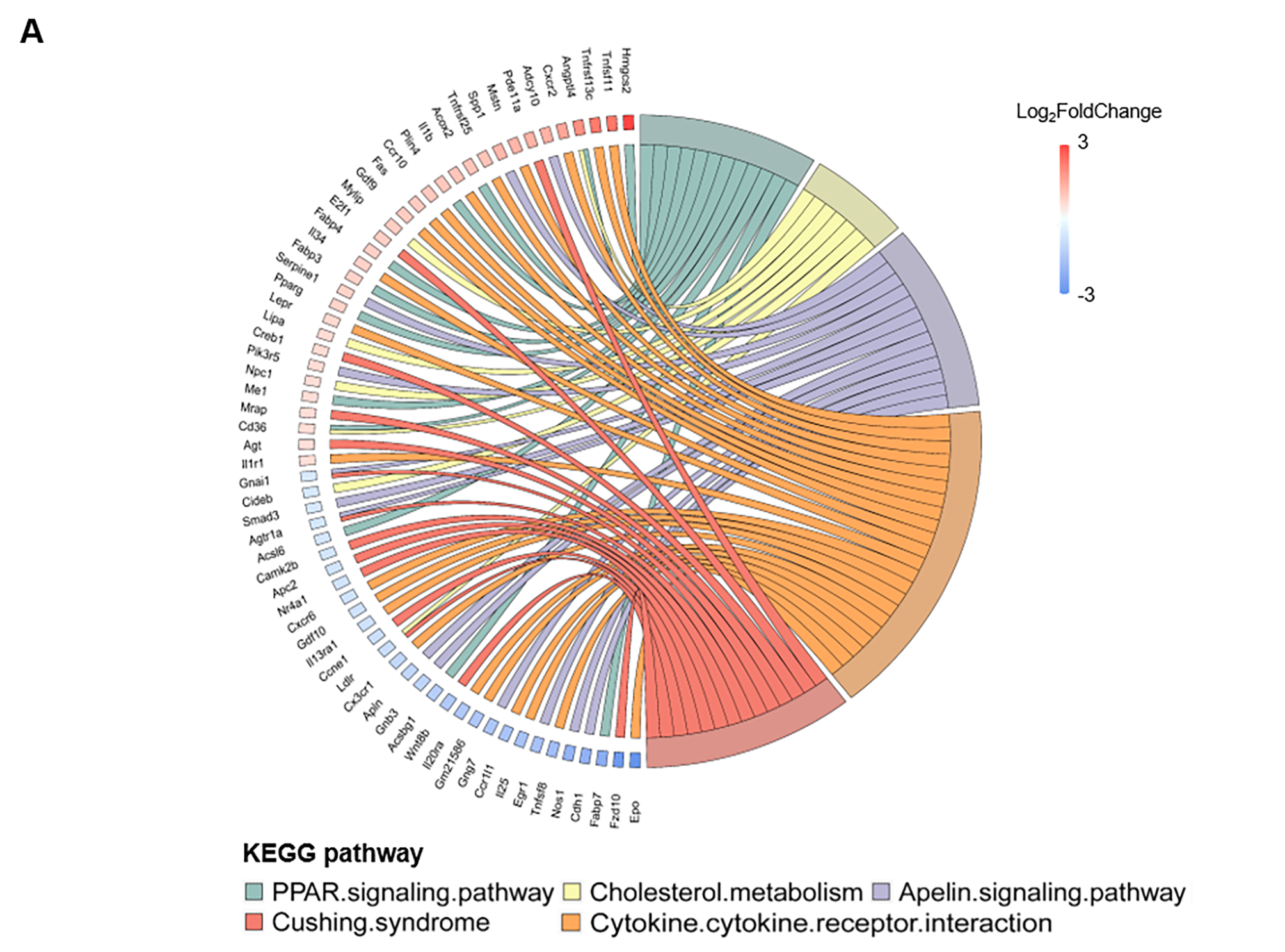

Supplement: Supplementary file 3 — Additional file 3: Figure S1. Top five KEGG pathway enrichment results in GSE180065. [file 12967_2023_4734_MOESM3_ESM.png]

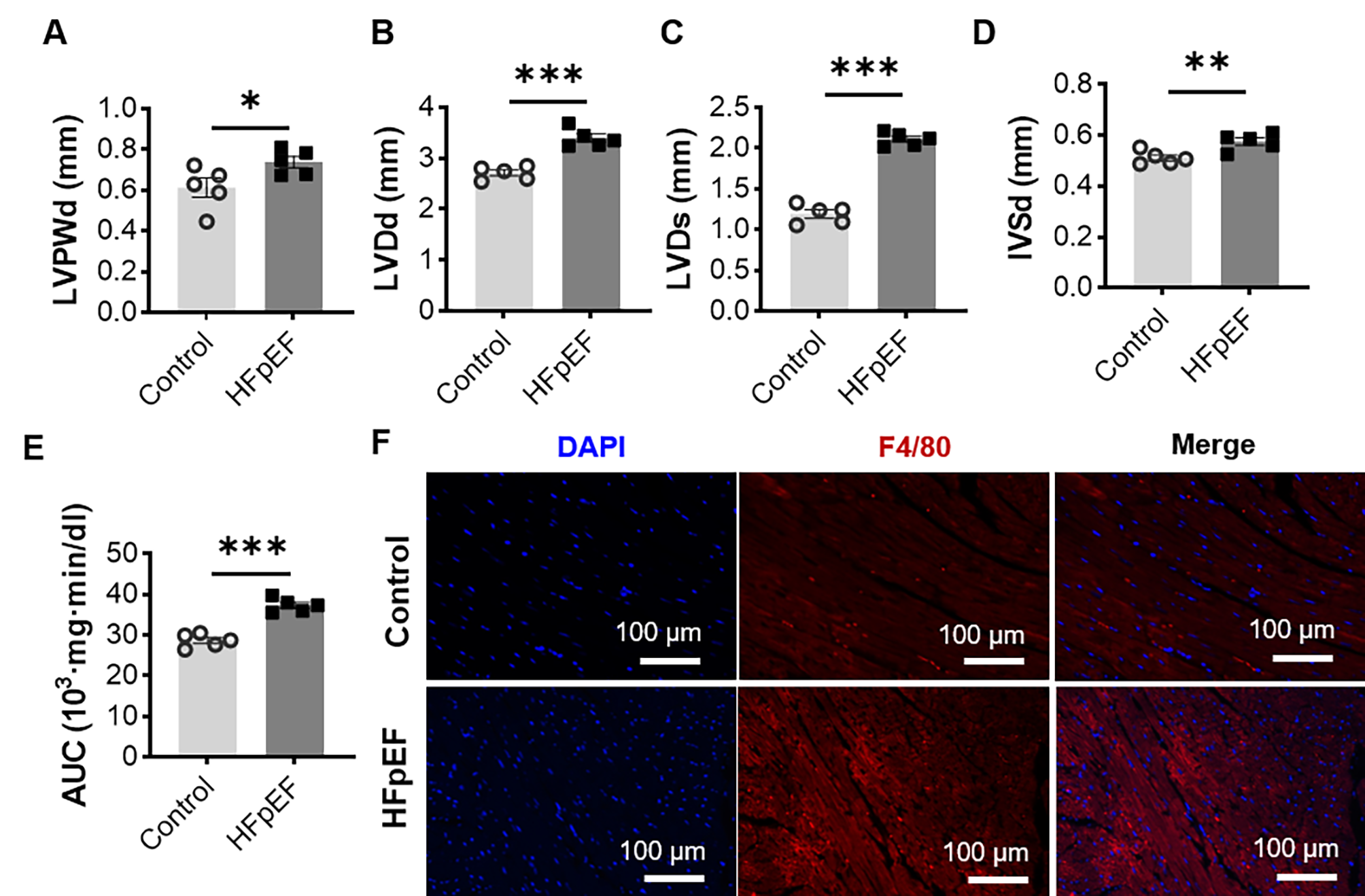

Supplement: Supplementary file 8 — Additional file 8: Figure S2. Additional material related to Figure 3. A LVPWd, n = 5 mice per group. B LVDd, n = 5 mice per group. C LVDs, n = 5 mice per group. D LVSd, n = 5 mice per group. E Area under the curve of the intraperitoneal glucose tolerance test experiment, n = 5 mice per group. F Representative images of immunohistochemical staining showing macrophage infiltration in the heart tissue, n = 5 mice per group. Statistical significance was calculated by Student’s t test; *P < 0.05, **P < 0.01, ***P < 0.001. [file 12967_2023_4734_MOESM8_ESM.png]

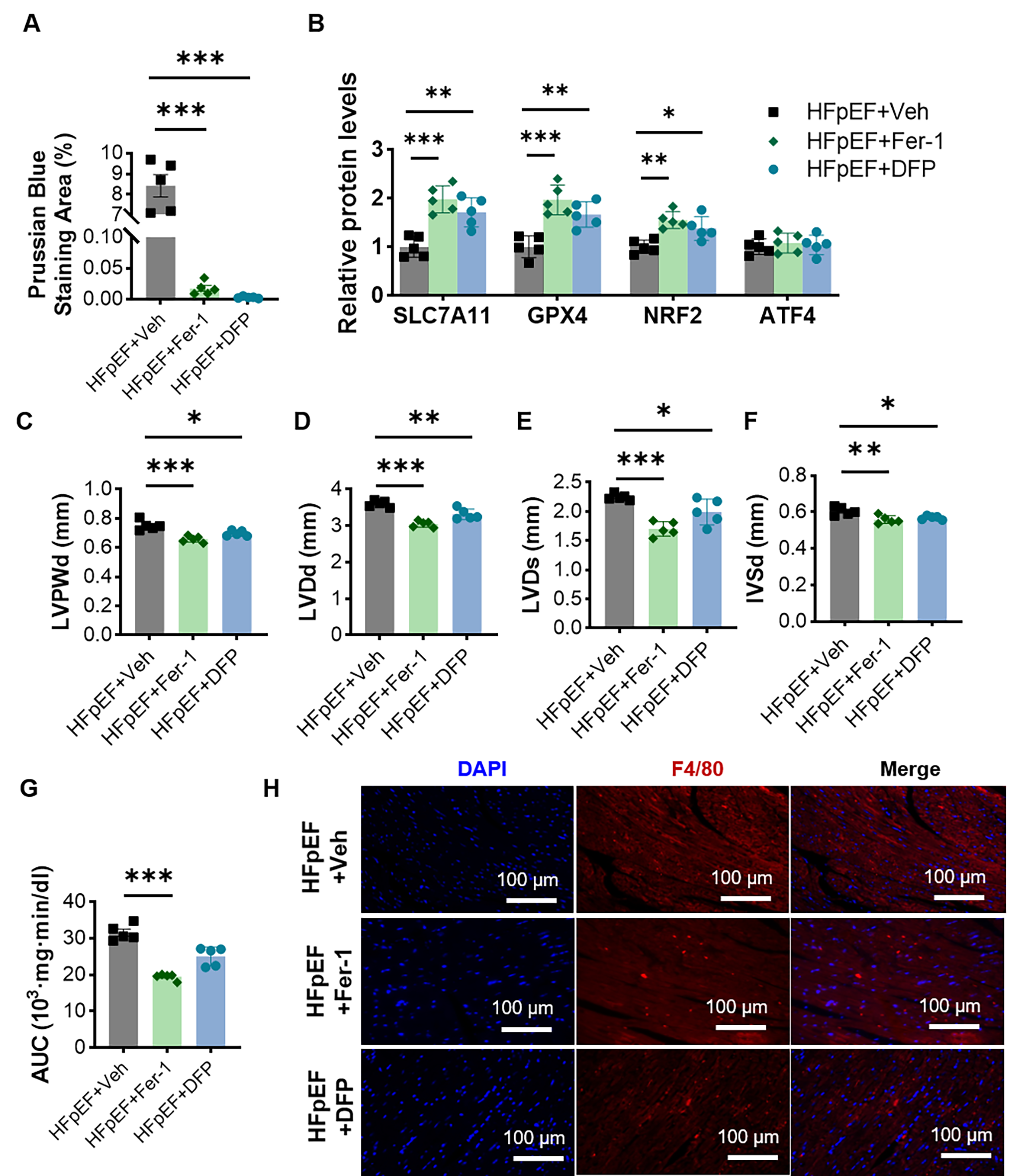

Supplement: Supplementary file 9 — Additional file 9: Figure S3. Fer-1 and DFP inhibit intramyocardial ferroptosis and ameliorate the HFpEF phenotype. A Percentage of Prussian blue-stained positive cells, n = 5 mice per group. B Average protein levels of SLC7A11, GPX4, NRF2 and ATF4 in mouse heart tissue, n = 5 mice per group. C LVPWd, n = 5 mice per group. D LVDd, n = 5 mice per group. E LVDs, n = 5 mice per group. F LVSd, n = 5 mice per group. (G) Area under the curve of the intraperitoneal glucose tolerance test experiment, n = 5 mice per group. H Representative images of immunohistochemical staining showing macrophage infiltration in the heart tissue, n = 5 mice per group. Statistical significance was calculated by one-way ANOVA; *P < 0.05, **P < 0.01, ***P < 0.001. [file 12967_2023_4734_MOESM9_ESM.png]
